# Renal sympathetic denervation for resistant hypertension: where do we stand after more than a decade

**DOI:** 10.1590/2175-8239-JBN-2018-0213

**Published:** 2020-01-10

**Authors:** Marco Antônio Peliky Fontes, Lucas Alexandre Santos Marzano, Carina Cunha Silva, Ana Cristina Simões e Silva

**Affiliations:** 1 Universidade Federal de Minas Gerais, Departamento de Fisiologia e Biofísica, Belo Horizonte, MG, Brasil.; 2 Universidade Federal de Minas Gerais, Faculdade de Medicina, Departamento de Pediatria, Belo Horizonte, Brasil.

**Keywords:** Hypertension, Sympathetic nervous system, Sympathectomy, Denervation, Renal hypertension, Hipertensão, Sistema Nervoso Simpático, Simpatectomia, Denervação, Hipertensão renal

## Abstract

Despite the current availability of safe and efficient drugs for treating hypertension, a substantial number of patients are drug-resistant hypertensives. Aiming this condition, a relatively new approach named catheter-based renal denervation was developed. We have now a clinically relevant time window to review the efficacy of renal denervation for treating this form of hypertension. This short review addresses the physiological contribution of renal sympathetic nerves for blood pressure control and discusses the pros and cons of renal denervation procedure for the treatment of resistant hypertension.

## Resumo

Em que pese a atual disponibilidade de medicamentos seguros e eficientes para o tratamento da hipertensão, um número significativo de pacientes sofre de hipertensão arterial resistente a tratamento medicamentoso. Em vista dessa condição, foi desenvolvida uma abordagem relativamente nova, denominada denervação renal por cateter. Dispomos atualmente de uma janela de tempo clinicamente relevante para analisar a eficácia da denervação renal no tratamento dessa modalidade de hipertensão. A presente revisão aborda a contribuição fisiológica dos nervos renais simpáticos no controle da pressão arterial e discute os prós e contras do procedimento de denervação renal no tratamento da hipertensão resistente.

## Introduction

Currently, hypertension is a global public health problem. In the United States, the 2017 Heart Disease and Stroke Statistics Update shows that about 85.7 million, or 34 percent, of American adults have high blood pressure^1^. In China, the adjusted prevalence of hypertension is 29.6%^2^. In Brazil, 36 million people are affected, an estimated prevalence of 32.5%, contributing directly or indirectly to 50% of deaths due to cardiovascular disease^3^. Therefore, the development of new pharmacological or non-pharmacological strategies for controlling blood pressure is extremely important.

Blood pressure is controlled, to a large extent, by sympathetic nerves, which are tonically active and define the sympathetic tone in heart, vessels, and kidneys^4^. Inactivation of specific central nervous system regions results in immediate fall in renal sympathetic nerve activity with consequent fall in blood pressure, and this effect is pronounced in the experimental model of essential hypertension^5^^,^ 6. The sympathetic innervation of the kidney plays an important role in all aspects of renal physiology, and renal sympathetic hyperactivity is considered central for human hypertension development and its progression^7^^-^ 9. In chronic kidney disease, renal nerves contribute to hypertension by increasing sympathoexcitation to other targets^10^.

Presently, numerous effective anti-hypertensive drugs are available, including antiadrenergic, diuretics, ACE inhibitors, angiotensin receptor blockers, calcium-channel blockers, and anti-renin drugs. However, a significant number of patients with essential hypertension are drug-resistant, i.e., patients that are unable to achieve goal blood pressure levels despite usage of 3 different antihypertensive agents at the appropriate dosage^11^.

**Figure 1:**
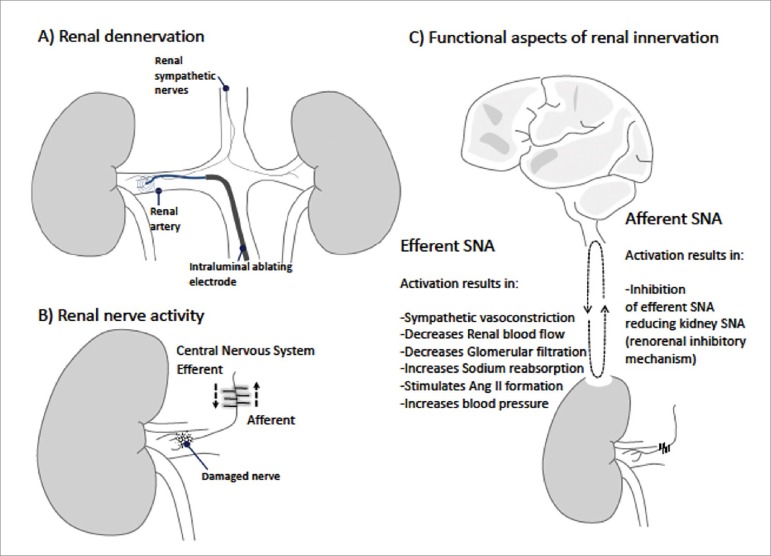
Renal denervation (A) alters the sympathetic cross-talk between kidneys and the central nervous system interrupting both efferent and afferent signaling (B and C). A) Radiofrequency energy (represented by dotted circles) is delivered into the lumen of renal arteries leading to thermal disruption of postganglionic sympathetic nerves directed to the kidney. B) Arrows / lines represent action potentials travelling along efferent and afferent fibers; this signaling is interrupted by renal denervation. C) Functional aspects of renal innervation involving intact efferent and afferent fibers signaling. Efferent activity changes kidney function and altered kidney function stimulates afferent signals leading to inhibition of efferent signaling. See text for details. SNA = sympathetic nerve activity.

The need for an alternative treatment for severe resistant hypertension led to the development of radiofrequency renal denervation. The procedure consists of delivering radiofrequency energy into the lumen of renal arteries leading to thermal disruption of postganglionic sympathetic nerves directed to the kidney^12^ (Figure 1A).

More than a decade after the first patient was treated with the radiofrequency catheter system, the efficacy of renal denervation is under consideration^12^^,^ 13. Findings of different clinical trials are quite different^14^ varying from a large reduction in ambulatory systolic blood pressure, reaching an average of -20 mmHg at 6 months as reported in early trials^15^^,^ 16 to an astonishing non-significant effect^17^. Of course, differences in blood pressure levels, medications, and procedures among trials might account for these discrepant results. However, a recent proof-of-concept trial for renal denervation published by Raymond Townsend and colleagues can bring additional light to this scenario. The results are modest but consistent (ambulatory systolic blood pressure −5.5 mmHg at 3 months), supporting the correlation between reduction in renal sympathetic tone and reduction in blood pressure^18^. It is important to highlight, however, that studies use a variety of techniques for renal sympathetic denervation (Table 1) and their different findings lead to different conclusions about the renal denervation, which increases the importance of this discussion. Thus, this is an appropriate time to review some physiological and clinical aspects of the procedure. The first part of this short review addresses the functional contribution of renal sympathetic nerves for blood pressure control. The second part discusses the pros and cons of renal denervation procedure for the treatment of resistant hypertension.

**Table 1:** Experimental and clinical studies on sympathetic renal denervation

Authors	Technique/ method	Species/Number	Result
Kandzari DE, Böhm M, Mahfoud F, et al, on behalf of the SPYRAL HTN-ON MED Trial Investigators	Renal Denervation	Humans/n=80	This trial has shown significant differences in favor of renal denervation reported between the groups 3 months after the procedure evaluated by 24-h ambulatory measurements. **(In favor)**
Azizi M, Schmieder, RE, Mahfoud F, et al, on behalf of the RADIANCE-HTN Investigators	Endovascular ultrasound renal denervation	Humans/n=146	The study concluded that the procedure was able to significantly reduce systolic and diastolic blood pressure between the groups at 2 months after the procedure evaluated by 24-h ambulatory measurements. **(In favor)**
Townsend RR, Mahfoud F, Kandzari DE, et al. On behalf of the SPYRAL HTN-OFF MED trial investigators (2017)	Renal Denervation	Humans/n=80	This trial has shown significant differences in favor of renal denervation reported between the groups in the 3 months change in 24-h ambulatory measurements. **(In favor)**
Bhatt DL, M.D, MPH, et al. For the SYMPLICITY HTN-3 Investigators (2014)	Renal artery denervation	Humans/n=535	This trial did not demonstrate a benefit of renal artery denervation on reduction in ambulatory BP in either the 24-h or day and night periods compared with sham. **(Against)**
Fink GD, Phelps JT. (2017)	Bilateral renal denervation	Rats/n=15	Data from this animal experiment did not identify a clinically useful way of predicting the magnitude of the blood pressure fall that occurs after renal denervation. **(Against)**
Salman IM, Hildreth CM, Phillips JK. (2017)	Vagal afferent stimulation	Rats/n=63	Data from this study provides direct insight into the role of the vagal afferent outflow in the regulation of cardiovascular function in chronic kidney disease in males and females **(In favor)**
Goodwill VS, Terrill C, Hopewood I, Loewy AD, Knuepfer MM (2017)	Infusion of isotonic or hypertonic NaCl	Rats/n=56	This experiment has shown that normal saline had little effect on afferent nerve activity, while hypertonic NaCl elicited an increase in renal afferent nerve activity. **(In favor)**
Zheng H, Patel KP (2017)	-	-	This study shows that an enhanced/altered afferent renal input to the paraventricular nucleus in disease conditions such as chronic heart failure and hypertension may be involved in producing elevated sympathetic nerve activity commonly observed in these disease states. **(In favor)**
Howden EJ, Esler JSLM, Levine BD (2017)	Endurance training	-	According to this review, endurance exercise training clearly lowers sympathetic activity in sympatho-excitatory disease states. It also influences many factors which may mediate a reduction in sympathetic activity However, the utility of endurance training as a countermeasure to alter sympathetic nerve activity in CKD patients remains to be determined **(Inconclusive)**
Olaf Grisk (2017)	Bilateral renal denervation	-	According to this review, even if new techniques were applied that may reduce the degree of renal reinnervation, the beneficial actions of renal denervation may still be offset by denervation supersensitivity. **(Against)**
Nishihara M, Takesue K, Hirooka Y (2017)	Bilateral renal denervation and bicuculline microinjection	Mice/n=101	According to this study, renal denervation has a sustained antihypertensive effect by increasing the urinary sodium excretion levels in the early phase and inhibiting SNA in association with augmented (In favor) GABA-ergic input into the paraventricular nucleus of the hypothalamus in the late phase in hypertensive mice with chronic kidney disease. **(In favor)**
Yao Y, Davis G, Harrison JC, Walker RJ, Sammut IA (2017)	Diabetes induction and bilateral renal denervation	Rats/n=27	Findings of this study support the conclusions that sympathetic tone is important in the pathophysiological development of hypertensive renal damage in diabetes. **(In favor)**
Veiga GL, Nishi EE, Estrela HF, Lincevicius GF, Gomes GN, Sato AYS, Campos RR, Bergamaschi CT (2017)	Induction of chronic kidney disease and total renal denervation	Rats/n=44	Data from this study suggest that hypertension, reduced renal function, and increased sympathoexcitation to other targets are at least partially driven by renal nerves in CKD. **(In favor)**

### Renal sympathetic innervation: physiological role of efferent and afferent fibers

Peripheral neural sympathetic outflow is a regionally differentiated process. This means that a stimulus can increase sympathetic activity in one or more organ or regions and decrease or produce no effect in others. Renal nerves contain both efferent and afferent nerve fibers, also called efferent or afferent renal nerves^19^^-^ 21. These nerves lie adjacent to the wall of renal arteries but they are not symmetrically distributed. Indeed, a deep understanding of the microanatomy of renal nerves is necessary for the best cardiovascular results produced by renal denervation^22^. A recent study in humans demonstrated that 1) the right renal artery has significantly higher innervation than the left, 2) the anterior and superior quadrants present higher innervation than the posterior and inferior quadrants, and 3) the distal third of the renal arteries are more innervated than the proximal segments^23^. Therefore, an efficient denervation removes the central sympathetic hyperactivity that will lead to consistent and durable blood pressure reduction.

The efferent fibers carry nerve impulses from the central nervous system towards the kidney influencing kidney function. The main neurotransmitter of the sympathetic efferent terminals is norepinephrine that acts on the vascular and nephron structures^20^. On the other hand, afferent or sensory fibers carry impulses originated in the kidney towards the central nervous system. The majority of the afferent sensory fibers are located in the renal pelvic wall^24^. The traffic of nerve signaling between the central nervous system and kidneys, and vice versa, is known as *renal nerve activity* (Figure 1B). Therefore, the renal nerve activity contains the *efferent renal sympathetic nerve activity* (ERSNA) and the *afferent renal nerve activity* (ARNA). In other words, the so called “*nerve activity*” is determined by the traffic of action potentials travelling from the brain to the kidney and from the kidney to the brain (Figure 1B). Efferent and afferent renal signals can be experimentally identified and measured in mammals^25^^-^ 27.

Renal afferent fibers respond to different stimuli, including chemical (e.g., inflammatory mediators), mechanical (e.g., increase in pelvic pressure), and nociceptive (e.g., kidney stones). It is generally accepted that some afferent signals exert a negative feedback on efferent sympathetic signals, constituting an autonomic inhibitory renorenal reflex (Figure 1C). Elevation of the intrarenal pressure increases afferent discharge and results in hypotension mediated by reduction in efferent sympathetic activity^28^. Activation of renal sensory afferent fibers by a high sodium diet decreases efferent sympathetic signals, which increases urinary sodium excretion to maintain sodium homeostasis^19^. Experiments in rats demonstrated that normal saline had little effect on afferent nerve activity, while hypertonic NaCl elicited an increase in renal afferent nerve activity^29^. On the other hand, experimental evidence shows that the afferent signaling resulting from renal injury increases efferent renal sympathetic activity leading to hypertension^30^. The enhanced or altered afferent renal input to the paraventricular nucleus in disease conditions may be involved in producing elevated sympathetic nerve activity^31^. In addition, evidence indicates that functional ablation of renal afferent fibers does not affect the regulation of arterial pressure in normal conditions but may play a role in the development of salt-sensitive hypertension^32^. In conclusion, there is a reciprocal interaction between efferent and afferent signals, and such interaction certainly depends on the stimulus and kidney condition.

In fact, the physiological contribution of the afferent signaling received little or no attention in the history of sympathetic denervation for hypertension treatment^33^^,^ 34. However, with the advent of radiofrequency renal denervation, the interest on renal afferent signaling for blood pressure control has been renewed^20^^,^ 26 and a great deal of information will come in the next decade.

Sympathetic efferent fibers are functionally specific in certain target organs. Consistent evidence indicates that this is the case for the kidney, where functionally specific efferent fiber groups could selectively innervate and control the main renal neuroeffectors; the vasculature, the tubules, and the juxtaglomerular granular cells^35^. Therefore, increases in renal sympathetic activity, 1) results in renal vasoconstriction, that consequently, 2) decreases renal blood flow, 3) decreases glomerular filtration rate, 4) increases renin release stimulating Ang II formation, and 5) decreases urinary sodium excretion (increases renal tubular sodium reabsorption)^35^.

### Renal sympathetic innervation in hypertension development

Hypertension is generally associated with sympathetic hyperactivity^36^ In several situations, hypertension has been attributed to sympathetic overactivity, which can be triggered by afferent signals originating from the kidney and resetting sympathetic tone by stimulation of hypothalamic centers. It has been also considered that ATP receptors contribute to renal vessel hypertrophy during Ang II-induced hypertension^37^. Indeed, Ang II induces rapid release of ATP from sympathetic nerves, acting prejunctionally^38^. This effect of Ang II was very intensively detected in Dahl salt-sensitive rats, suggesting that ATP release from renal sympathetic nerves may contribute to the development of salt-sensitive hypertension^39^.

Chronic activation of renal sympathetic nerves, in both animal and human studies, is critical in the pathogenesis and perpetuation of resistant hypertension. Consequently, renal denervation has emerged as a therapeutic option in disease states characterized by central sympathetic overactivity^40^^,^ 41. Renal sympathetic nerves enhance dendritic cell activation, T-cell infiltration, and kidney tissue damage in the development of hypertension^42^. Furthermore, renal sympathetic nerves contribute to hypertension by means of sodium retention, stimulation of renin secretion, and augmentation of sympathetic activity induced by the central nervous system. Based on all these mechanisms, renal denervation has been indicated for the treatment of hypertension, mostly the resistant forms^43^.

### Renal sympathetic denervation: pros and cons

Some important questions should be discussed in regard to renal sympathetic denervation: Is the procedure proven to be effective in both animals and humans? Under what conditions can it help? What are the risks? What are the side effects in the short or long term?

#### Efficiency

Pre-clinical studies with several animal models of hypertension showed reduction in blood pressure following renal sympathetic denervation^44^^,^ 45 ^,^ 46 ^,^ 47. For instance, in experimental studies with elderly mice with high systolic blood pressure, there was a drop in blood pressure after the procedure and this effect persisted for many weeks^44^. On the other hand, in younger mice, the fall in blood pressure after denervation was not sustained^44^. In different experimental models of hypertension, renal denervation reduced baseline blood pressure^48^^,^ 45 ^,^ 46 ^,^ 47, with concomitant decrease in plasma levels of epinephrine, norepinephrine, renin activity, angiotensin II, and aldosterone^49^. In addition, studies have also suggested that renal denervation improves mechanisms in other target organs including left-ventricular function^50^, endothelial nitric oxide bioavailability^51^, glucose metabolism, and insulin sensitivity^52^^,^ 53. Moreover, after renal denervation, heart-failure patients presented improvements in symptoms and exercise capacity^54^. In this regard, Ukena and colleagues found that the cardiorespiratory response in patients submitted to exercise is not affected after renal denervation^55^. The benefits of regular physical activity to reduce cardiovascular risk are well-known^56^. Therefore, improved organ function and preserved exercise capacity after renal denervation can result in better control of hypertension.

However, there is a clear dilemma in proving the efficacy of renal sympathetic denervation based on the results of clinical trials with hypertensive patients. Some clinical trials found the procedure effective, while others questioned its efficacy^57^. Authors who speak against the treatment rely on the results of the Symplicity HTN-3 trial^12^. This trial did not find a benefit of renal artery denervation in regard to the reduction of ambulatory blood pressure (BP) in either the 24-hour or day and night periods compared with controls^17^. However, when this trial is critically analyzed, we can find some flaws in its execution. Indeed, the technique of denervation used in the study was inconsistent to determine the effectiveness of the procedure^12^. Retrospective analysis of stored angiographic records of all radio frequency energy applications showed that in 74% of patients, energy application did not achieve a full circumference of the renal artery, which was a mandatory protocol requirement and should have been bilateral^12^^,^ 58. Meanwhile, the SPYRAL HTN-OFF MED study (n=38 in the intervention group and n=42 in the sham-operated control group) indicated that the renal sympathetic denervation was effective for the treatment of patients with mild to moderate hypertension (24-h systolic blood pressure (SBP) -5 mm Hg (95% CI -9.9 to -0.2; *p*=0.0414), 24-h diastolic blood pressure (DBP) -4.4 mm Hg)^59^. However, a critical analysis of that trial suggests that after treatment the patient would still need to take oral antihypertensive therapy to achieve adequate systolic blood pressure levels. In addition, patients with mild to moderate elevation of blood pressure are not recommended for this kind of treatment.

More recently, two trials have investigated the efficacy of renal denervation, the SPYRAL HTN-ON MED^60^ and the RADIANCE-HTN SOLO^61^

The SPYRAL HTN-ON MED proof-of-concept randomized trial, published in May 2018, aimed to evaluate the safety and efficacy of catheter-based renal denervation compared to sham control for the treatment of uncontrolled hypertension^60^. Patients were submitted to renal angiography and randomly allocated to sham control or renal denervation groups. The primary outcome was blood pressure change from baseline, based on ambulatory blood pressure measurements assessed at 6 months. The Symplicity Spyral multielectrode renal denervation catheter (Medtronic, Galway, Ireland) and the Symplicity G3 renal denervation RF generator (Medtronic, Minneapolis, MN, USA) were used to provide circumferential radiofrequency ablation in a spiral pattern in the four quadrants of the renal artery and of branch vessels between three and eight mm in diameter. The control group was submitted to a sham. The study had a small number of subjects (38 in the renal denervation group and 42 in the control group) and did not measure the efficiency of renal nerve ablation, although the number of ablations per patient and the technique were similar to those reported in the SPYRAL HTN-OFF MED trial. The study reported a significantly change in BP after 6 months of the procedure in both 24-h SBP and DBP measurements. SBP had a reduction of -7.4 mmHg (95% CI -12.5 to -2.3; *p*=0.0051) and DBP of -4.1 mmHg (95% CI -7.8 to -0.4; *p*=0.0292). The findings suggested an effect in patients adherent and non-adherent to antihypertensive medications but due to the small sample size the difference between adherent and non-adherent could not be evaluated. Nevertheless, adherence was similar in both groups, and ambulatory BP measurements were obtained only following witnessed pill ingestion in all patients. However, similar to the SPYRAL HTN-OFF-MED trial, the procedure did not result in adequate levels of SBP, leading patients to continue using oral antihypertensive therapy^60^. This study is still ongoing and will be completed on December 2022 (available at https://clinicaltrials.gov/ct2/show/NCT02439749).

The RADIANCE-HTN SOLO trial, also published in 2018, used an endovascular ultrasound renal denervation system in patients with uncontrolled hypertension and in patients with mild to moderate hypertension. Patients were randomized (1:1) to undergo renal denervation with the Paradise system (ReCor Medical, Palo Alto, CA, USA) or a sham procedure consisting of renal angiography only. Renal nerve ablation was obtained by a minimum of two sonications of 7 seconds each in the main branch of the right and left renal artery, separated longitudinally by 5 mm. The study included 74 patients for renal denervation and 72 for sham procedure. Renal denervation significantly reduced SBP and DBP (- 6.3 mmHg, 95% CI -9.4 to -3.1, *p*=0.0001 and -2.6 mmHg, 95% CI -4.6 to -0.6, *p*= 0.01, respectively). The study evaluated only the safety and efficiency of the procedure for two months, in which patients remained off antihypertensive medications. Results were good, but still not enough to permanently stop oral antihypertensive therapy^61^ This trial is still ongoing until August 2021 (available at https://clinicaltrials.gov/ct2/show/NCT02649426).

Table 1 summarizes experimental studies and clinical trials evaluating the effect of renal sympathetic denervation for control of arterial hypertension.

#### Risks of the procedure and side effects

The potential risks of the procedure include internal hemorrhage, arterial stenosis, and aneurysms, however rarely reported in the literature^12^.

Concerning side effects of the treatment, there are some data to be considered. Previously, there was a dogma^62^ that the renal sympathetic nerve would only act in the renal blood flow under pathological conditions or by an external stimulation, not occurring under physiological conditions. However, it is now proven that the sympathetic renal nerve has a role also under physiological conditions^46^ and therefore, side effects of the treatment should be expected. For example, renal nerves provide protection against ischemic injuries by limiting ischemia and reperfusion effects on organs susceptible to damage. This effect seems to be impaired after the renal sympathetic denervation treatment^63^.

Other studies indicated that there might be side effects related to cardiac baroreflex, mostly during physical exercise and after sudden postural changes^64^. In these situations, a sudden drop in blood pressure can occur, which may lead to fainting and/or capillary hypoperfusion^64^. Recent evidence also suggests that the procedure can affect the compensatory hemodynamic responses to hemorrhage. Singh and colleagues reported that renal denervation reversed the hypertension in sheep with chronic kidney disease and improved renal function. In response to hemorrhage, the fall in mean arterial pressure was greater in the denervated than the intact group. The increase of heart rate and plasma renin activity were significantly attenuated in sheep submitted to renal denervation when compared with intact sheep^65^.

Besides that, the used technique allows natural sympathetic reinnervation. This nerve regrowth is a troubling condition and is associated with an escape phenomenon, which contributes to the return of hypertension^66^^,^ 67. Functional nerve regrowth was also observed in animal trials^68^. It was also suggested that nerve regrowth occurs in native human kidneys after renal transplantation^69^. Due to increased organ sensitization to adrenalin attributed to upregulation of adrenergic receptors, nerve regrowth after denervation leads to the escape phenomenon, since blood pressure increases even more in comparison to levels prior the surgical procedure^67^. In animal studies, rats develop renal norepinephrine super sensitivity and functional reinnervation after perivascular renal denervation^68^. This complication is critical for patients with resistant hypertension and chronic kidney disease.

### Concluding remarks

Despite animal studies frequently reporting beneficial effects of renal sympathetic denervation for the control of hypertension, results of clinical trials are still somewhat disappointing^17^^,^ 70 ^,^ 71. The efficacy of renal denervation for reducing blood pressure in drug-resistant hypertension is currently inconclusive, although it appears to work in some patients^72^. The main limitation is that, even after an apparently successful renal denervation, patients still need to use oral anti-hypertensive medications. In addition, side effects related to the procedure seem not to be a relevant issue, but they should still be taken into account.

It should be mentioned, however, that there are three ongoing clinical trials. The Global Clinical Study of Renal Denervation With the Symplicity Spyral™ Multi-electrode Renal Denervation System in Patients With Uncontrolled Hypertension in the Absence of Antihypertensive Medications (SPYRAL PIVOTAL - SPYRAL HTN-OFF MED) started on June 2015 and will be completed on December 2022 (available at https://clinicaltrials.gov/ct2/show/NCT02439749). The purpose of the study was to test the hypothesis that renal denervation decreases blood pressure and is safe in the absence of antihypertensive medications. Primary outcomes include acute and chronic safety by evaluating incidence of major adverse events from baseline to 1 month post-procedure and changes in SBP measured by 24-hour ambulatory BP monitoring from baseline to 3 months post-procedure. The RADIANCE-HTN is a randomized, double-blind, sham controlled, two cohort studies (TRIO and SOLO) designed to show the efficacy and document the safety of the Paradise Renal Denervation System in two distinct populations of hypertensive subjects (available at https://clinicaltrials.gov/ct2/show/NCT02649426). Subjects with essential hypertension controlled by 1 or 2 antihypertensive medications or uncontrolled will be included in the RADIANCE Solo cohort, whereas subjects with resistant hypertension on a minimum of 3 antihypertensive medications will be included in the RADIANCE Trio cohort. The primary outcome is reduction in average daytime ambulatory SBP from baseline to 2 months after the procedure. The RADIANCE-HTN trial started on March 2016 and will be completed on August 2021. The RADIANCE II Pivotal Study is a randomized trial to test the effectiveness and safety of the Paradise System in subjects with stage 2 hypertension on 0-2 medications at the time of consent (available at https://clinicaltrials.gov/ct2/show/NCT03614260). Prior to randomization, subjects should be hypertensive in the absence of hypertension medication. Primary outcomes are incidence of major adverse events from baseline to 30 days post-procedure and changes in average daytime ambulatory SBP from baseline to 2 months post-procedure. The study started on December 2018 and will be completed on October 2024. Although focused only on short-term effects, these ongoing trials will provide more data on the efficacy and safety of renal denervation.

Finally, the development of devices or surgical procedures for hypertension treatment that interfere with sympathetic nervous system has renewed the importance of sympathetic nerve activity in relation to human cardiovascular control. In this regard, the American Physiological Society recently published a guideline article aiming to provide standard recommendations for measuring sympathetic activity in humans and other mammals^73^. Optimal measurement of sympathetic activity in humans via microneurography can avoid misleading data and incorrect conclusions related to the efficacy of the procedure for a specific group of patients. Further clinical trials adopting standard protocols for the measurement of sympathetic activity in patients submitted to renal sympathetic denervation are needed for the final establishment of the role of this procedure for drug-resistant hypertension.
